# Limited usefulness of neurocognitive functioning indices as predictive markers for treatment response to methylphenidate or neurofeedback@home in children and adolescents with ADHD

**DOI:** 10.3389/fpsyt.2023.1331004

**Published:** 2024-01-12

**Authors:** Anna Kaiser, Pascal M. Aggensteiner, Hilario Blasco Fontecilla, Tomas Ros, Eric Acquaviva, Yohan Attal, Tobias Banaschewski, Sarah Baumeister, Elisa Bousquet, Aurore Bussalb, Marie Delhaye, Richard Delorme, Renate Drechsler, Allison Goujon, Alexander Häge, Louis Mayaud, Konstantin Mechler, Caroline Menache, Olivier Revol, Friederike Tagwerker, Susanne Walitza, Anna Maria Werling, Stéphanie Bioulac, Diane Purper-Ouakil, Daniel Brandeis

**Affiliations:** ^1^Department of Child and Adolescent Psychiatry and Psychotherapy, Central Institute of Mental Health, Medical Faculty Mannheim/Heidelberg University, Mannheim, Germany; ^2^UNIR Health Sciences School and Medical Center and UNIR-itei, CIBERSAM, Madrid, Spain; ^3^Department of Neuroscience, Campus Biotech CISA-Université de Genève, Genève, Switzerland; ^4^Child and Adolescent Psychiatry Department and Child Brain Institute, Robert Debré Hospital, Assistance Publique-Hôpitaux de Paris and Universite Paris Cite, Paris, France; ^5^myBrain Technologies, Paris, France; ^6^Unit of Child and Adolescent Psychiatry (MPEA1), CHU Montpellier-Saint Eloi Hospital, University of Montpellier, Montpellier, France; ^7^Mensia Technologies, Paris, France; ^8^Child and Adolescent Psychiatry, Erasme Academic Hospital, Université Libre de Bruxelles, Bruxelles, Belgium; ^9^Department of Child and Adolescent Psychiatry and Psychotherapy, University Hospital of Psychiatry Zurich, University of Zurich, Zurich, Switzerland; ^10^Clinique des Grangettes, Chêne-Bougeries, Geneva, Switzerland; ^11^Unit of Child and Adolescent Psychiatry, Hospices civils de Lyon, Hôpital Femme Mère Enfant, Bron Cedex, France; ^12^SANPSY, USR 3413, CNRS, Bordeaux, France; ^13^Clinique du Sommeil, CHU Pellegrin, Bordeaux Cedex, France; ^14^Development and Trajectories, INSERM CESP U 1018 Psychiatry, Montpellier, France; ^15^CESP, INSERM U 1018, Paul Brousse Hospital, Villejuif, France; ^16^Neuroscience Center Zurich, University of Zurich and ETH Zurich, Zurich, Switzerland; ^17^Zurich Center for Integrative Human Physiology, University of Zurich, Zurich, Switzerland

**Keywords:** neurocognitive functioning, executive functions, ADHD, predictive marker, treatment marker, methylphenidate, neurofeedback

## Abstract

**Introduction:**

Earlier studies exploring the value of executive functioning (EF) indices for assessing treatment effectiveness and predicting treatment response in attention-deficit/hyperactivity disorder (ADHD) mainly focused on pharmacological treatment options and revealed rather heterogeneous results. Envisioning the long-term goal of personalized treatment selection and intervention planning, this study comparing methylphenidate treatment (MPH) and a home-based neurofeedback intervention (NF@Home) aimed to expand previous findings by assessing objective as well as subjectively reported EF indices and by analyzing their value as treatment and predictive markers.

**Methods:**

Children and adolescents (*n* = 146 in the per protocol sample) aged 7–13 years with a formal diagnosis of an inattentive or combined presentation of ADHD were examined. We explored the EF performance profile using the Conners Continuous Performance Task (CPT) and the BRIEF self-report questionnaire within our prospective, multicenter, randomized, reference drug-controlled NEWROFEED study with sites in five European countries (France, Spain, Switzerland, Germany, and Belgium). As primary outcome for treatment response, the clinician-rated ADHD Rating Scale-IV was used. Patients participating in this non-inferiority trial were randomized to either NF@home (34–40 sessions of TBR or SMR NF depending on the pre-assessed individual alpha peak frequency) or MPH treatment (ratio: 3:2). Within a mixed-effects model framework, analyses of change were calculated to explore the predictive value of neurocognitive indices for ADHD symptom-related treatment response.

**Results:**

For a variety of neurocognitive indices, we found a significant pre-post change during treatment, mainly in the MPH group. However, the results of the current study reveal a rather limited prognostic value of neurocognitive indices for treatment response to either NF@Home or MPH treatment. Some significant effects emerged for parent-ratings only.

**Discussion:**

Current findings indicate a potential value of self-report (BRIEF global score) and some objectively measured neurocognitive indices (CPT commission errors and hit reaction time variability) as treatment markers (of change) for MPH. However, we found a rather limited prognostic value with regard to predicting treatment response not (yet) allowing recommendation for clinical use. Baseline symptom severity was revealed as the most relevant predictor, replicating robust findings from previous studies.

## 1 Introduction

Attention-deficit/hyperactivity disorder (ADHD) is a neurodevelopmental disorder characterized by pervasive inattention, hyperactivity, and impulsivity resulting in significant impairment including social and educational disadvantage (1–4). With a prevalence of approximately 5%, it is one of the most common psychiatric disorders in children and adolescents (5). Further, in around 60% of cases, the disorder persists into adulthood with 15–20% of adults (still) meeting the full diagnostic criteria, therefore, constituting a major public health problem throughout the lifespan (6). Symptoms typically emerge early during childhood and associated difficulties become most apparent in (later) school years when more attention, impulse control and higher-order executive functioning (EF) skills are required (7). Therefore, early identification of symptoms and adequate early intervention strategies are important for altering the developmental course of the disorder and prevent negative (long-term) outcomes (8–10).

Earlier studies exploring cognitive-functioning alterations in ADHD patients found that they often present with deficits regarding attentional processing, response inhibition, and further executive control processes (11). Typically, these deficits are reflected by lower accuracy (more errors) and higher variability of objective task-performance for ADHD patients compared to typically developing peers. Thereby, symptoms of impulsivity have been linked to higher rates of commission errors, while symptoms of inattention are typically associated with higher rates of omission errors as well as slower reactions times (RT), and a higher RT variability (12).

However, results on neuropsychological differences between patients diagnosed with ADHD and typically developing peers are rather heterogeneous and significant findings only reveal small to moderate effect sizes (12, 13), questioning the diagnostic value of neuropsychological indices for diagnostic purpose. Further, results from previous studies are not convincing regarding the specificity for ADHD diagnosis. Instead, findings indicate a rather low usefulness of those neuropsychological indices for reliably differentiating between ADHD and patients with other neurodevelopmental disorders (14–16). Therefore, neuropsychological indices are (currently) not recommended as a stand-alone marker indicative of the diagnosis of ADHD (17–20).

Even if neurocognitive impairments are not diagnostic for ADHD, they contribute to reduced school, family-life and social functioning in ADHD (21), and should therefore be interpreted as relevant mental-health assessment characteristics and important treatment targets (22). Previous studies already showed effects of pharmacological (23–28) as well as non-pharmacological (29, 30) treatments on (higher-order, but also “lower”) cognitive-functioning processes in ADHD (31). These findings highlight a potential use of those indices as treatment markers, and their relevance for understanding the (developmental) course of the disorder (21, 32). With regard to pharmacological treatment options, results from earlier studies reveal significant effects of psychopharmacological medication on a variety of neuropsychological outcome measures (e.g., stimulant medication reduces RT variability) (33). For example, Kawabe et al. (34) showed a significantly improved accuracy in children with ADHD on a working-memory test (one-back task), and significantly fewer errors, anticipatory errors, and shorter reaction times after methylphenidate (MPH) treatment (34). Further, Miklós and colleagues found that treatment-naïve children with ADHD showed weaker performance on EF measures than either medicated ADHD patients or typically developing peers (35). For non-pharmacological interventions, a recent meta-analysis found that among others, especially cognitive training, and EF-specific treatments might have beneficial effects on EF in children and adolescents with ADHD (29). For Neurofeedback (NF) as another non-pharmacological treatment option, Bink et al. (36) revealed improved outcomes of attention and motor speed, with faster processing times and with medium to large effect sizes for a combination of treatment-as-usual (TAU) and NF as well as TAU alone, but no improvements for higher EFs (36). Comparing MPH and NF treatment (as well as physical activity), Geladé et al. (37) found superior effects of stimulant medication over theta/beta ratio (TBR-)NF to improve neurocognitive functioning. Thereby, (positive) effects were higher in the MPH group compared to the NF group on attention, inhibition, and impulsivity as reflected by faster stop signal reaction times and lower commission and omission error rates (37). For the Continuous Performance Task (CPT), which has been broadly used in ADHD, Heinrich and colleagues (2004) found a decrease in impulsivity errors after 25 sessions of slow-cortical potentials (SCP-)NF training in a group of 13 children with ADHD (seven to 13 years). However, in a later larger (*N* = 77) study, no differences were found between SCP-NF and the control condition for commission errors but for hit rates (38). Another study using the CPT as well as the BRIEF as a subjectively-rated clinical scale for assessing higher-order EF deficits (39) within a substantially larger sample of children with ADHD (*n* = 104) compared NF, cognitive training and a control condition. The authors found that children in the NF group showed significant improvement compared to the control group on the objective CPT measures and on all BRIEF scores. There was no significant difference between the cognitive training and control groups. Correlations between changes in neurocognitive functioning indices and clinical symptom improvement were significant but weak probably due to a focus on clinical scales as relevant study outcomes rather than functional outcomes as primary measures of success. However, the latter might be important markers of treatment efficacy due to their translational value and relation to functional activities of daily living (10).

Further, a few promising findings suggest some potential of neuropsychological testing indices for predicting response to different ADHD treatment options (pharmacological and non-pharmacological) (40–42). With regard to pharmacological treatment options, Johnstone and colleagues found performance on a Go/No-Go task being one of the most important variables for classifying MPH responders versus non-responders (43). Further, van der Oord et al. (42) found that low levels of prepotent response inhibition are associated with worse response to treatment with MPH (42). However, these studies are characterized by small samples and substantial methodological heterogeneity. Furthermore, those studies primarily focused on prediction of treatment response to pharmacological therapy options (mainly, MPH and atomoxetine). A recent systematic review highlighted the potential usefulness of objectively-measured cognitive-task performance indices for treatment planning, especially with regards to predicting treatment response to stimulant medication, and emphasizes a lack of research on response to non-pharmacological, psychosocial interventions (44). Consequently, there is a need of further studies including larger, well-characterized samples for identifying predictors of treatment effectiveness of pharmacological as well as non-pharmacological interventions in ADHD patients with the aim to enhance treatment outcomes (45, 46), and for approaching the long-term goal of personalized treatment selection and intervention planning.

For the treatment of ADHD in children and adolescents, various pharmacological as well as non-pharmacological treatment options exist (10, 47). Besides pharmacotherapy, European guidelines recommend psychosocial treatment options within a multimodal treatment approach. With regard to pharmacotherapy, a substantial amount of randomized controlled trials (RCTs) as well as meta-analyses already demonstrated robust effects of methylphenidate (MPH), with immediate-release and long-acting stimulants showing substantially larger effects compared to non-stimulant medications such as atomoxetine (48, 49). Further RCTs and systematic quantitative reviews showed the efficacy of behavioral interventions, with moderate to even large effect sizes, especially in unblinded ratings (50–52). Importantly, NF interventions were in the focus of research in recent years, with meta-analytical findings resulting in small to moderate effect sizes, and effects showing up, most consistently, for standard training protocols (53, 54) and in unblinded ratings (55, 56). However, probably blinded ratings often result in small and non-significant findings questioning the effectiveness of NF for ADHD treatment. Therefore, studies are warranted focusing on NF-treatment individualization within a personalized medicine framework exploring mediators and predictors for treatment response (e.g., explicitly addressing setting intensity and patient subgroup characteristics). Studies directly comparing pharmacological treatment options with NF, indicated inferiority of NF training compared to MPH (61, 62). For example, Meisel et al. (61) explored a small sample of *n* = 23 children and young adolescents between 7 and 14 years of age either receiving MPH or NF treatment. Both groups showed similar ADHD symptom reductions with regards to the primary outcome for both, parent and teacher ratings. For functional impairment, symptom reductions were only revealed for the parent ratings. In their 12-week trial comparing MPH and NF for children newly diagnosed with ADHD, Sudnawa et al. (62) found large effect sizes for MPH treatment and moderate effect sizes for the NF training group; however, for teacher ratings, significant effects were only obtained for the MPH group, indicating relevant rater differences with regard to the evaluation of treatment response. The authors concluded that NF might be a promising alternative for children who do not respond to pharmacological treatment or experience significant adverse events (AEs) related to psychopharmacological therapies. Here, neurocognitive indices might provide reliable (additional) information with regard to the assessment of treatment effectiveness, as they represent objective measures excluding rater biases.

In recent years, digital interventions including home-based treatment options (for home-based application) became more and more prominent due to their ease of accessibility, probably lower costs, and possibly higher acceptance rates among patients. Especially in ADHD and younger patient populations, digital treatment options with a gaming character might enhance treatment motivation and compliance, consequently leading to higher treatment effectiveness. Therefore, a promising approach to enhance NF-training effects might be an implementation as a digital intervention for home-based training. For example, Shin et al. (63) were able to show that a smart-tablet based NF training might improve cognitive function in children with (subclinical) levels of inattention symptoms (63). Within our NEWROFEED trial, analyses related to the primary objective of testing for non-inferiority of a home-based NF training (NF@Home) compared to MPH in children and adolescents (aged 7–13 years) revealed that both treatment groups showed significant pre-post improvements in core ADHD symptoms and in a broader range of problems, but robust rejection of non-inferiority at the group level, with twice as high uncontrolled effect sizes in the MPH group (*d* = 2.03) compared to the NF@Home group (*d* = 0.89) for the primary outcome (64).

Within our NEWROFEED study (64, 65), a secondary objective was to identify predictors that might inform individualized treatment selection within a personalized medicine framework. Thereby, a focus was on exploring common and differential treatment effects for the optimized pharmacological (MPH) and the personalized intense non-pharmacological (NF@Home) treatment options within the study. A special emphasis was on objective neuropsychological tests and self-report scales assessing higher-order EF skills. Based on previous literature, a further aim was to explore differential treatment effects on those indices as well as associations of changes in those indices with changes on the level of clinical outcomes. Therefore, children with ADHD were assessed before (and after) receiving NF training at home (NF@home: SMR or TBR training) or MPH. We explored their neuropsychological performance profile and EF skills using the Conners Continuous Performance Task (CPT-3) and the Behavior Rating Inventory of Executive Functioning (BRIEF) questionnaire, respectively, and assessed the prognostic value of indices derived from these measures for predicting treatment response. The predictive value was analyzed separately for ratings by clinicians, important caregivers (parents) and teachers.

## 2 Materials and methods

### 2.1 Study design

Within the prospective, multicenter, randomized, reference drug-controlled NEWROFEED study (for details, see protocol paper) (65), children between 7 and 13 years of age with a formal diagnosis of an inattentive or combined presentation of ADHD were included. Patients were recruited between August 2016 and September 2017 in nine centers across five European countries (France, Spain, Switzerland, Germany, and Belgium). The diagnosis of ADHD was made by a clinician using the Kiddie-SADS (K-SADS) (2), a semi-structured interview with the child and the parents. Children were eligible if they had already received previous treatment for ADHD (i.e., psychoeducation), if they had a wireless internet connection at home, and if their parents and themselves gave signed informed consent (or children’s assent according to local requirements) (65).

Patients were randomized in one of two treatment groups subsequently receiving either Neurofeedback training at home (NF@Home group) or methylphenidate (MPH group) using a 3:2 randomization ratio. Hereby, the allocation sequence was computer-generated (using SAS software v9.4). During study participation, there were eight visits over a total duration of 3 months: pre-inclusion visit, inclusion visit (D0), four discovery (NF group) or four titration visits (MPH group), an intermediate visit (D60), and a final visit (D90). For a detailed description of measurements at all assessments, see protocol paper (65). Outcomes of the study were rated by clinicians, parents, and teachers. The investigator, the clinician rating the scales, and the parents were all unblinded.

The study was registered in the US National Institute of Health ClinicalTrials.gov under number #NCT02778360.^1^ The Research Ethics Boards at each of the participating centers approved the study.

### 2.2 Study interventions

#### 2.2.1 NF@home group

A medical-grade EEG device (Mensia Koala^TM^) with 8 AgCl electrodes (Fpz, Fz, F3, F4, Cz, C3, C4 and Pz) was used for the NF training. For each participant, the investigator calibrated the device during an initial qEEG session that also identified individualized alpha peak frequency (iAPF) to determine individualized EEG frequency bands and the TBR for group/training assignment to either SMR or TBR training (66). Depending on their theta/beta ratio (TBR) assessed via electroencephalography (EEG) before treatment (D0), patients in the NF@Home group were assigned to one of two different standardized NF-training protocols: SMR training or TBR training. For a (pre-treatment) TBR < 4.5 μV, patients trained the SMR-up-regulation. For a TBR > 4.5 μV, patients trained TBR-down-regulation.

Each NF@Home training session consisted of five 4 min-long ‘active’ NF blocks (with real-time feedback) and two 2.5 min-long ‘transfer’ blocks (with only intermittent feedback). After initiation visits at the clinic (with no specific instructions given by the investigator), the family took the NF device home for the duration of the treatment period.

The first treatment phase consisted of 16 to 20 sessions (4 per week), followed by the mid-assessment visit (D60). The second treatment phase was of similar length and ended with the final assessment visit (D90). Further details about the study protocol, including the reinforcement schedule and content, were published elsewhere (65).

#### 2.2.2 MPH group

After an open titration period of 3 weeks starting with 10 mg of extended-release MPH per day, there was a maximum possible dose of 60 mg/day during the treatment period lasting for 2 months. The optimal dose had to remain stable during this period.

### 2.3 Study outcomes/measures

For assessing treatment response, the clinician-rated ADHD Rating Scale-IV (ADHD-RS-IV) (67, 68) was used as the primary outcome. Furthermore, the parent-and teacher-rated ADHD-RS-IV were assessed as secondary endpoints.

To measure cognitive and higher-order EF, the Behavior Rating Inventory of Executive Function (BRIEF) (69) and the Conners Continuous Performance Test 3 (CPT-3) (70) were implemented.

### 2.4 Data analysis

Longitudinal ANCOVAs within a mixed-model framework were calculated to analyze predictive effects of neurocognitive and EF indices on treatment response to intense pharmacological (MPH) as well as non-pharmacological (NF@home) treatment options, and to replicate and extend our published non-inferiority results (64). Separate analyses were calculated for symptom change scores between D0, D60, and D90 (total, inattention, and hyperactivity/impulsivity) from clinician, parent and teacher ratings. As predictors, the following continuous variables/indices were included from the BRIEF and the CPT, respectively: the BRIEF global score, the CPT Hit reaction time (raw score for correct targets in ms), the CPT reaction time variability (SDs of RTs in ms), the CPT omission errors (raw sum score), and the CPT commission errors (raw sum score). All analyses were controlled for the effects of baseline ADHD symptom levels, study center, age, and IQ. Furthermore, interaction terms between the predictors and the treatment group were included to explore differential predictive effects for each treatment within the study design. Furthermore, univariate ANOVAS (using change scores), t-tests for paired samples, and correlations were calculated for further and *post-hoc* analyses. The alpha level was set to *p* < 0.05.

## 3 Results

### 3.1 Sample description

The per-protocol sample for the neurocognitive/EF prediction analyses consists of *N* = 146 children and adolescents with ADHD (see Supplementary Figure S1 for CONSORT FLOW diagram). 87 patients were randomized into the NF@Home treatment group, with 72 being allocated to the SMR training, and 15 to the TBR training condition. 59 patients were randomized to MPH treatment. Descriptive statistics for the three treatment groups are displayed in Table 1.

**Table 1:** Sample descriptives.

	NF@Home SMR training group	NF@Home TBR training group	MPH group	Group difference
N	72	15	59	–
Age, M (SD)	10.49 (1.48)	9.34 (1.04)	9.78 (1.79)	SMR > TBR/MPH, *p* = 0.02
IQ, M (SD)	109.61 (15.90)	111.40 (18.42)	105.39 (14.88)	*n.s.*
Sex (% female)	23.61%	20.00%	15.25%	*n.s.*
D0 Clinician-rated ADHD total score	33.7 (8.46)	35.1 (8.29)	36.3 (8.57)	*n.s.*

There was no significant difference between the three groups with regard to clinician-rated ADHD baseline symptom severity [*F*(2, 143) = 1.50, *p* > 0.05, *n.s.*] or IQ [F(2, 143) = 1.53, *p* > 0.05, *n.s.*]. However, there was a significant age difference between the groups [F(2, 143) = 3.96, *p* = 0.02], with significantly older patients in the SMR group compared to the (small) TBR and the MPH groups [SMR-TBR: t(85) = 2.24, *p* = 0.03; SMR-MPH: t(129) = 2.18, *p* = 0.03; TBR-MPH: t(72) = −0.89, *p* = 0.38].

### 3.2 Description of neuropsychological, higher-order cognitive functioning indices

Descriptive statistics for the neuropsychological, higher-order cognitive indices (across all participants) can be found in Table 2.

**Table 2:** Descriptives for neuropsychological, higher-order cognitive functioning indices.

Index	N	Mean	SD	Min	Max
CPT omission errors	148	7.18	9.86	0.00	69.44
CPT commission errors	148	52.58	19.51	0.00	93.06
CPT Hit RT (ms)	148	489.31	102.91	296.47	873.72
CPT Hit RT variability (ms)	142	105.97	73.28	16.25	437.81
BRIEF global score	147	189.11	28.54	108.00	251.00

### 3.3 Between- and within-treatment effects: ADHD symptom change D0-D60-D90

#### 3.3.1 Clinician ratings (D0-D60-D90)

**Figure 1:**
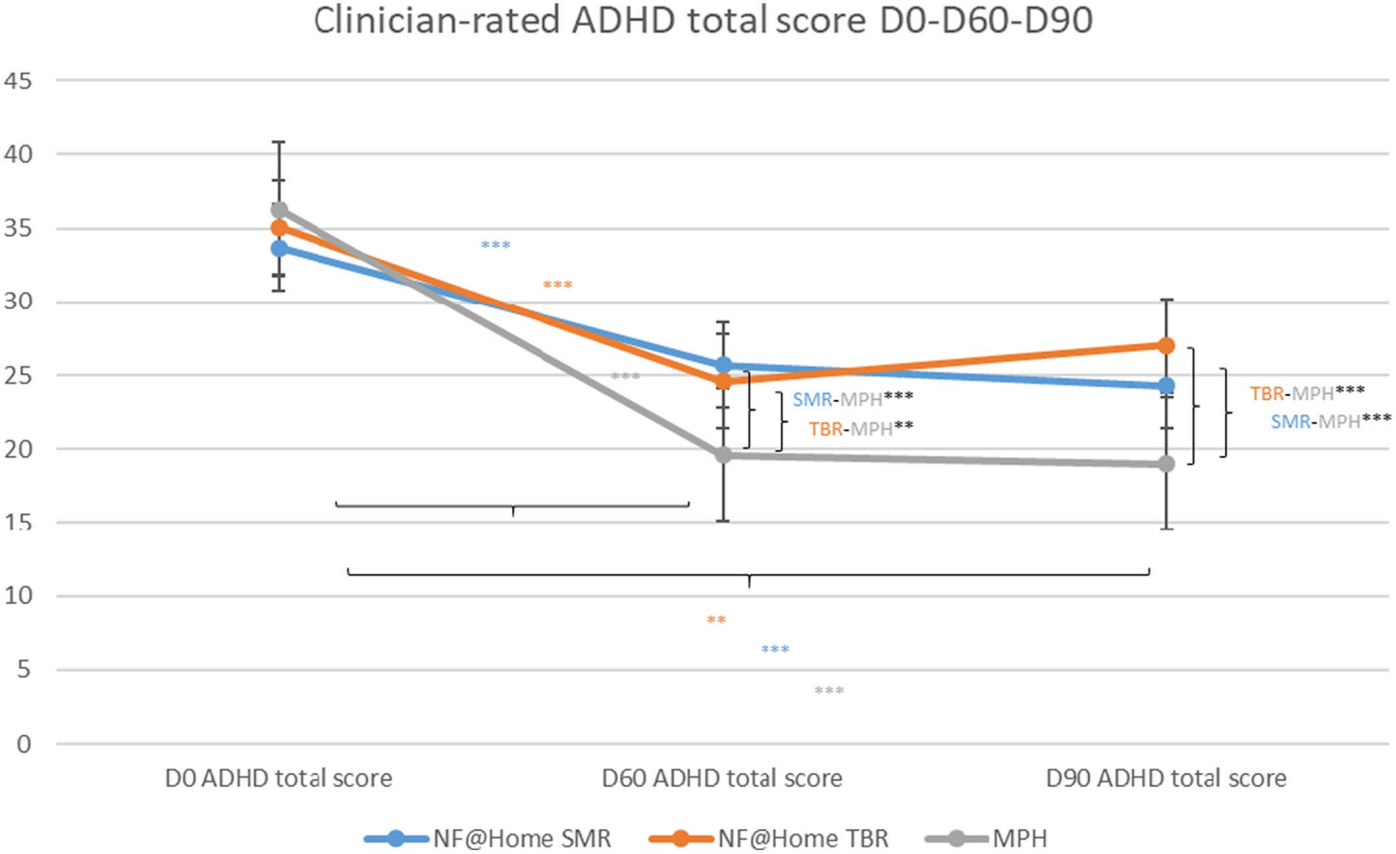
Clinician-rated ADHD total scores D0-D60-D90. *p < 0.05, **p < 0.01, ***p < 0.001.

With regard to the ADHD total score, a significant between-group difference was found for MPH compared to SMR as well as for MPH compared to TBR at D60 [SMR-MPH: 7.23, SE = 1.18, 95% CI 4.89–9.57, t(133) = 6.11, *p* < 0.001, *d* = 0.86; TBR-MPH: 6.84, SE = 1.39, 95% CI 3.03–10.60, t(133) = 3.55, *p* < 0.01, *d* = 0.81] as well as at D90 [SMR-MPH: 6.55, SE = 1.49 95% CI 3.60–9.50, t(133) = 4.39, *p* < 0.001, *d* = 0.86; TBR-MPH: 9.87, SE = 2.44, 95% CI 5.04–14.70, t(133) = 4.05, *p* < 0.001, *d* = 1.17]. There was no significant difference between the two NF@Home groups, neither for D60 nor for D90 (*p* > 0.05; *n.s.*). The same pattern of results was obtained for both subscores, ADHD inattention and hyperactivity/impulsivity (see Supplementary Tables S2–S4; Figure 1).

For all treatment groups, within-group results revealed statistically significant symptom reductions with regard to the ADHD total score for all groups from D0 to D60 [SMR: −8.77, SE = 1.07, 95% CI −10.90−(−6.66), t(133) = −8.24, *p* < 0.001, *d* = −1.04; TBR: −9.16, SE = 1.95, 95% CI −13.00−(−5.30), t(133) = −4.69, *p* < 0.001, *d* = −1.08; MPH: −16.00, SE = 1.20, 95% CI −18.40−(−13.60), t(133) = −13.30, *p* < 0.001, *d* = −1.89], as well as to D90 [SMR: −10.10, SE = 1.23, 95% CI −12.50−(−7.65), t(133) = −8.21, *p* < 0.001, *d* = −1.19; TBR: −6.76, SE = 2.36, 95% CI −11.40−(−2.09), t(133) = −2.86, *p* < 0.01, *d* = −0.80; MPH: −16.60, SE = 1.37, 95% CI −19.30−(−13.90), t(133) = −12.10, *p* < 0.001, *d* = −1.97]. A similar pattern of results was obtained for the ADHD subscores (see Supplementary Tables S2–S4), however, for the hyperactivity/impulsivity dimension, a significant symptom reduction in the TBR group was only obtained for D60 [−3.37, SE = 1.14, 95% CI −5.61-(−1.12), t(133) = −2.96, *p* < 0.01, *d* = −0.51], but not for D90 (*p* > 0.05, *n.s.*).

#### 3.3.2 Parent ratings (D0-D60-D90)

**Figure 2:**
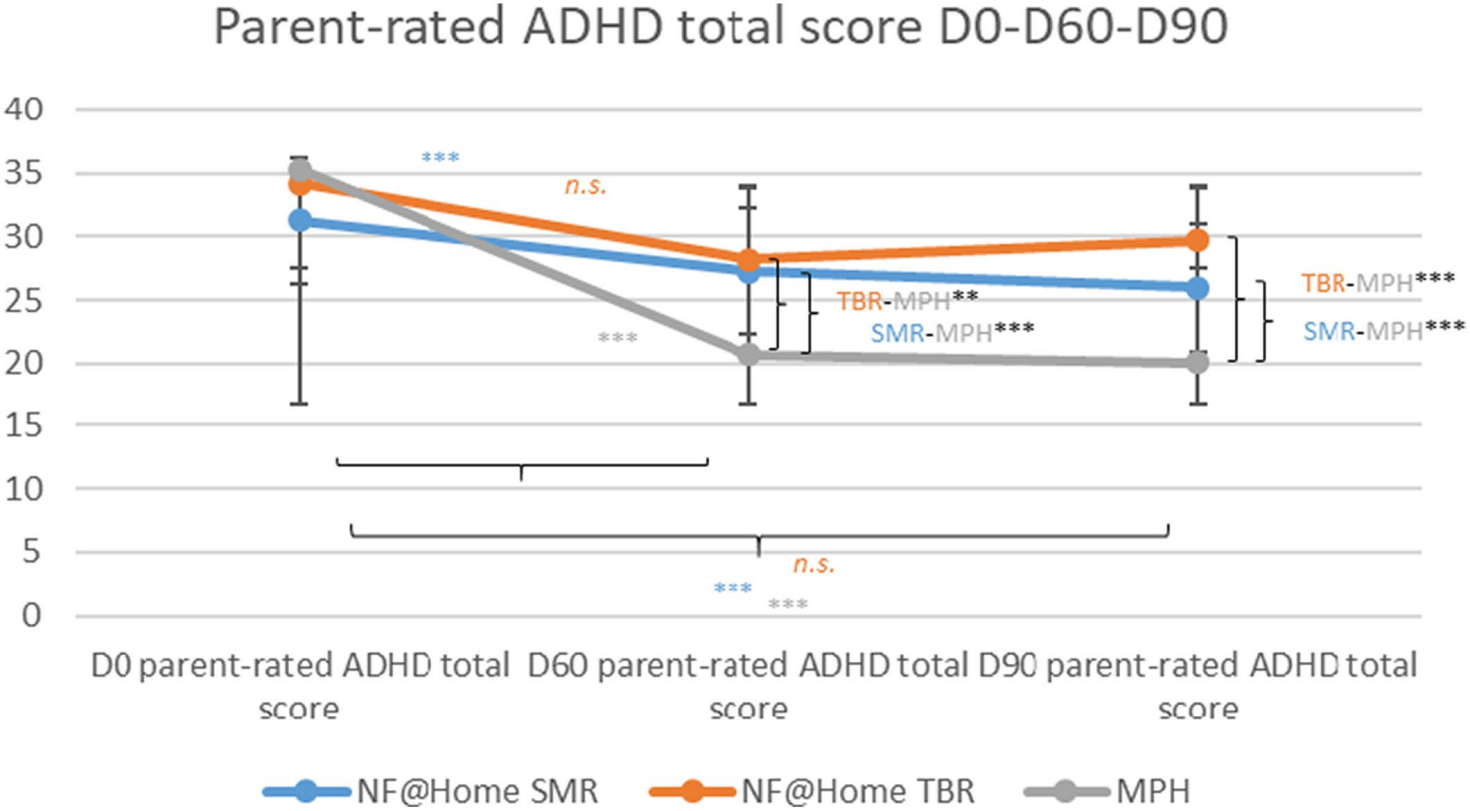
Parent-rated ADHD total scores D0-D60-D90. *p < 0.05, **p < 0.01, ***p < 0.001.

With regard to the between-group effects for the ADHD total score, the same pattern of results was obtained as for the clinician ratings [D0-D60: SMR-MPH: 68.23, SE = 1.47 95% CI 5.33–11.10, t(132) = 5.62, *p* < 0.001, *d* = 0.82; TBR-MPH: 8.55, SE = 2.35, 95% CI 3.89–13.20, t(132) = 3.63, *p* < 0.01, *d* = 0.85; SMR-TBR: *n.s.*; D0-D90: SMR-MPH: 7.75, SE = 1.74 95% CI 4.30–11.20, t(132) = 4.45, *p* < 0.001, *d* = 0.77; TBR-MPH: 10.5, SE = 2.81, 95% CI 4.94–16.10, t(132) = 3.74, *p* < 0.001, *d* = 1.05; SMR-TBR: *n.s.*]. The same pattern of results was obtained for both subscores, ADHD inattention and hyperactivity/impulsivity (see Supplementary Tables S5–S7; Figure 2).

Within-group results revealed statistically significant symptom reductions similar to the clinician ratings with regard to the ADHD total score for the SMR and MPH groups from D0 to D60 [SMR: −4.57, SE = 1.30, 95% CI −7.14−(−2.00), t(132) = −3.51, *p* < 0.001, *d* = −0.46; MPH: −12.80, SE = 1.46, 95% CI −15.70−(−9.91), t(132) = −8.76, *p* < 0.001, *d* = −1.28], as well as to D90 [SMR: −5.57, SE = 1.44, 95% CI −8.40−(−2.70), t(132) = −3.85, *p* < 0.001, *d* = −0.5 5; MPH: −13.30, SE = 1.62, 95% CI −16.50−(−10.10), t(132) = −8.20, *p* < 0.001, *d* = −1.33], but not for the TBR group (D60 and D90: *p* > 0.05, *n.s.*). All within-effects turned out to be significant for the inattention subscale. For the hyperactivity/impulsivity subscale, there was no statistically significant effect in the TBR group, neither for D60 nor for D90 (see Supplementary Tables S5–S7).

#### 3.3.3 Teacher ratings (D0-D90)

**Figure 3:**
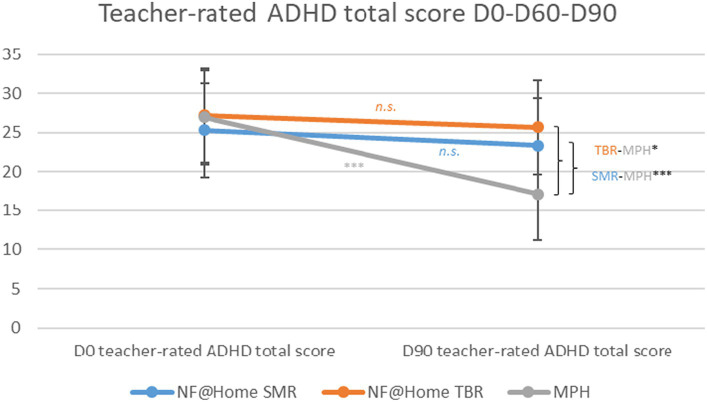
Teacher-rated ADHD total scores D0-D90. *p < 0.05, **p < 0.01, ***p < 0.001.

In the teacher ratings, there was a significant between-group effect between MPH compared to SMR, as well as MPH compared to TBR for the ADHD total score [SMR-MPH: 7.68, SE = 2.04, 95% CI 3.61–11.70, t(74) = 3.76, *p* < 0.001, *d* = 0.65; TBR-MPH: 8.01, SE = 3.31, 95% CI 1.41–14.60, t(74) = 2.42, *p* < 0.05, *d* = 0.68] as well as for the inattention subscale. In line with the results obtained from clinician and teacher rating, there was no significant difference between the two NF@Home groups (*p* > 0.05; *n.s.*). For the hyperactivity/impulsivity dimension, only the difference between the MPH and SMR groups showed a statistically significant effect [3.40, SE = 1.02, 95% CI 1.37–5.44, t(76) = 3.33, *p* < 0.01, *d* = 0.46; see Supplementary Tables S8–S10; Figure 3].

Within-group analyses only revealed significant effects for the MPH group from D0 to D90 for the ADHD total score [MPH: −10.00, SE = 1.42, 95% CI −12.90−(−7.19), t(74) = −3.05, *p* < 0.001, *d* = −0.85], as well as the inattention subscale [MPH: −5.84, SE = 0.82, 95% CI −7.47−(−4.21), t(75) = −7.15, *p* < 0.001, *d* = −0.98] and the hyperactivity/impulsivity subscale [MPH: −4.27, SE = 072, 95% CI −5.70−(−2.84), t(76) = −5.95, *p* < 0.001, *d* = −0.58]. No statistically significant symptom reductions were found for the two NF@Home groups, neither for the ADHD total score nor for any of the two subscales (*p* > 0.05; *n.s.*; see Supplementary Tables S8–S10).

### 3.4 Changes in neuropsychological/EF indices D0-D90

#### 3.4.1 SMR group

A statistically significant improvement between D0 and D90 was revealed for the BRIEF global score, with lower values at D90 [m_D0_ = 187.12, sd_D0_ = 30.12; m_D90_ = 174.19, sd_D90_ = 30.71; m_diff_ = 12.93, sd_diff_ = 24.16, t(67) = 4.41, *p* < 0.001]. There were no statistically significant changes between D0 and D90 for any of the other neuropsychological, higher-order cognitive functioning indices for the SMR group (*p* > 0.05; *n.s.*).

#### 3.4.2 TBR group

There were no statistically significant changes between D0 and D90 for any of the neuropsychological, higher-order cognitive functioning indices for the TBR group (*p* > 0.05; *n.s.*).

#### 3.4.3 MPH group

Statistically significant improvement between D0 and D90 were obtained for the following neuropsychological, higher-order cognitive functioning indices: statistically significant less CPT omission errors at D90 [m_D0_ = 9.02, sd_D0_ = 12.69; m_D90_ = 5.43, sd_D90_ = 9.98; m_diff_ = 3.60, sd_diff_ = 12.32, t(58) = 2.24, *p* = 0.30], less commission errors [m_D0_ = 53.55, sd_D0_ = 20.39; m_D90_ = 41.43, sd_D90_ = 19.47; m_diff_ = 12.12, sd_diff_ = 14.92, t(58) = 6.24, *p* < 0.001], faster CPT hit RTs [m_D0_ = 503.27, sd_D0_ = 101.75; m_D90_ = 479.13, sd_D90_ = 97.65; m_diff_ = 24.14, sd_diff_ = 56.56, t(58) = 3.28, *p* = 0.002], lower CPT Hit RT variability [m_D0_ = 114.28, sd_D0_ = 69.63; m_D90_ = 76.51, sd_D90_ = 58.67; m_diff_ = 37.77, sd_diff_ = 73.18, t(53) = 3.79, *p* < 0.001], and lower BRIEF global score [m_D0_ = 191.27, sd_D0_ = 26.35; m_D90_ = 164.91, sd_D90_ = 30.57; m_diff_ = 26.36, sd_diff_ = 28.97, t(55) = 6.81, *p* < 0.001].

### 3.5 Association between change in neuropsychological/EF indices and clinical improvement (D0-D90)—across all treatments

A few associations between changes in neurocognitive/EF indices and clinical improvement (D0-D90) turned out significant: the change in CPT commission errors and the ADHD total score rated by parents (*r* = 0.22, *p* = 0.008), the change in CPT Hit RT variability and ADHD total score rated by clinicians (*r* = 0.19, *p* = 0.02) as well as by parents (*r* = 0.17, *p* = 0.05), and the change in the BRIEF global score rated by clinicians (*r* = 0.52, *p* < 0.001), parents (*r* = 0.72, *p* < 0.001), as well as teachers (*r* = 0.23, *p* = 0.04).

### 3.6 Predictive effects of neuropsychological indices (D0) on ADHD treatment response (D0-D90)

#### 3.6.1 Clinician ratings

For clinician ratings, there was no significant predictive effect of any of the neuropsychological/EF indices on ADHD symptom change. However, a robust predictive effect was found for ADHD symptom severity at baseline for change on the ADHD total score [t(114) = −5.36, *p* < 0.001], as well as on both ADHD subscores [inattention: t(114) = −6.97, *p* < 0.001; hyperactivity/impulsivity: t(114) = −7.86, *p* < 0.001].

#### 3.6.2 Parent ratings

With regards to the parent ratings, there was a significant predictive interaction effect for treatment group and CPT omission errors on change in the ADHD total score [t(113) = 2.29, *p* = 0.02]. This effect was driven by the inattention subscale [t(113) = 2.34, *p* = 0.02], with (more) symptom improvement being associated with a lower CPT omission score at D0 in the small TBR group (*r* = 0.52, *p* = 0.05); results in the SMR and MPH groups showed only small and non-significant associations in the same direction (*p* < 0.05, *n.s.*). In addition, a significant predictive interaction effect was found for treatment group and the BRIEF global score [t(113) = 2.00, *p* < 0.05], with more symptom improvement being linked to higher BRIEF global score values at D0 (indicating a higher level of executive dysfunction) in the SMR (*r* = −0.29, *p* = 0.02) and MPH groups (*r* = 0.51, *p* < 0.001) and no association in the small TBR group (*p* > 0.05, *n.s.*). For the hyperactivity/impulsivity subscale, a significant predictive main effect was obtained for CPT hit RT [t(113) = 2.09, *p* = 0.04], with slower CPT hit RTs at D0 being associated with higher improvement/symptom change. However, *post-hoc* correlations yielded no significant effects in any of the treatment groups (*p* > 0.05, *n.s.*). Furthermore, a robust predictive effect was found for ADHD symptom severity at baseline for change on the ADHD total score [t(113) = −3.47, *p* < 0.001], as well as on both ADHD subscores [inattention: t(113) = −5.49, *p* < 0.001; hyperactivity/impulsivity: t(113) = −5.06, *p* < 0.001].

#### 3.6.3 Teacher ratings

For teacher ratings, there was no significant predictive effect of any of the neuropsychological/EF indices on ADHD symptom change. However, a robust predictive effect was found for ADHD symptom severity at baseline for change on the ADHD total score [t(57) = −4.00, *p* < 0.001], as well as on both ADHD subscores [inattention: t(58) = −50, *p* < 0.001; hyperactivity/impulsivity: t(59) = −4.31, *p* < 0.001].

### 3.7 Effects of baseline symptom levels on treatment response

Significant effects of baseline symptom levels on ADHD symptom change (D0-D60-D90) were obtained for all three raters (clinicians, parents, and teachers).

**Figure 4:**
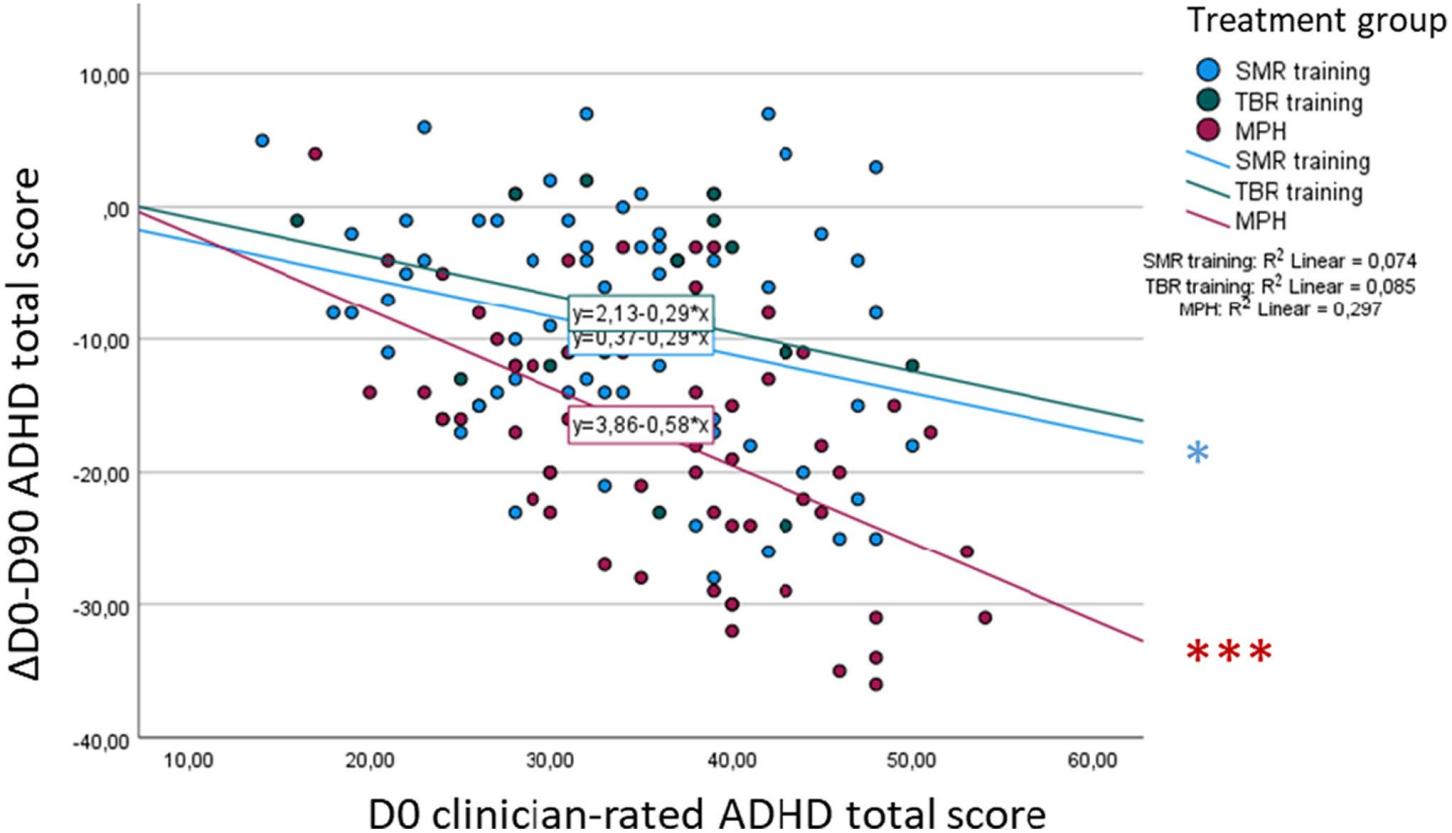
Association between baseline (D0) ADHD total score and D0-D90 symptom change—clinician rating. *p < 0.05, **p < 0.01, ***p < 0.001.

There was a significant effect of the baseline on clinician-rated ADHD total score symptom change [*F*(1, 140) = 13.37, *p* < 0.001]. *Post-hoc* analyses revealed a significant negative association, with higher baseline symptom levels being linked to a higher treatment response, only in the SMR (*r* = −0.27, *p* = 0.02) and MPH groups (*r* = −0.55, *p* < 0.001), with no significant differences in associations between all three groups (Figure 4).

**Figure 5:**
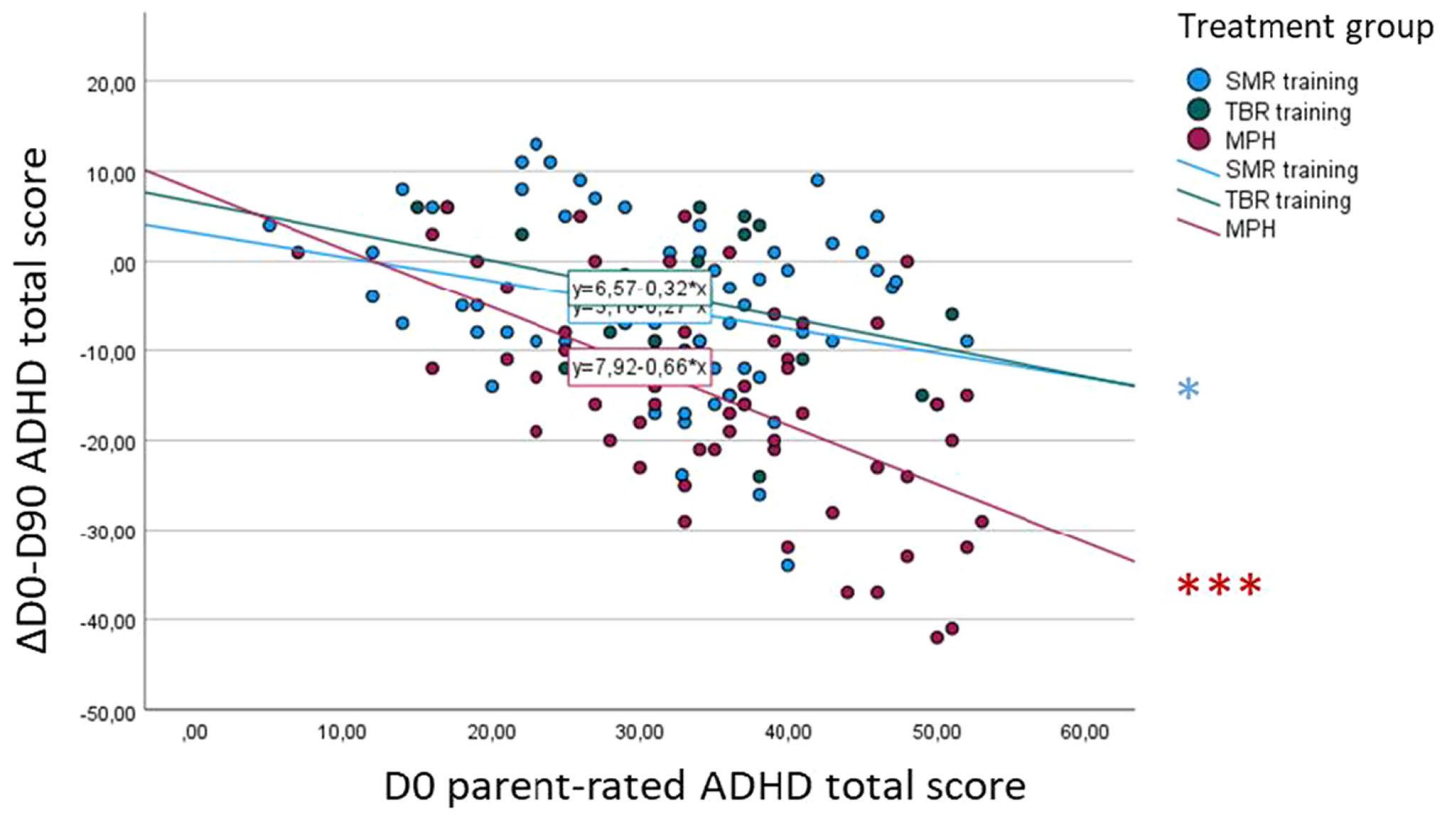
Association between baseline (D0) ADHD total score and D0-D90 symptom change—parent rating. *p < 0.05, **p < 0.01, ***p < 0.001.

Also, there was a significant effect of baseline on parent-rated ADHD total score symptom change [*F*(1, 138) = 16.30, *p* < 0.001]. Within *post-hoc* analyses, we could identify significant negative correlations only in the SMR (*r* = −0.28, *p* = 0.02) and MPH groups (*r* = −0.59, *p* < 0.001), with a significantly higher association in the MPH group compared to the SMR group (diff = 0.31, *z* = 2.15, *p* = 0.03; Figure 5).

**Figure 6:**
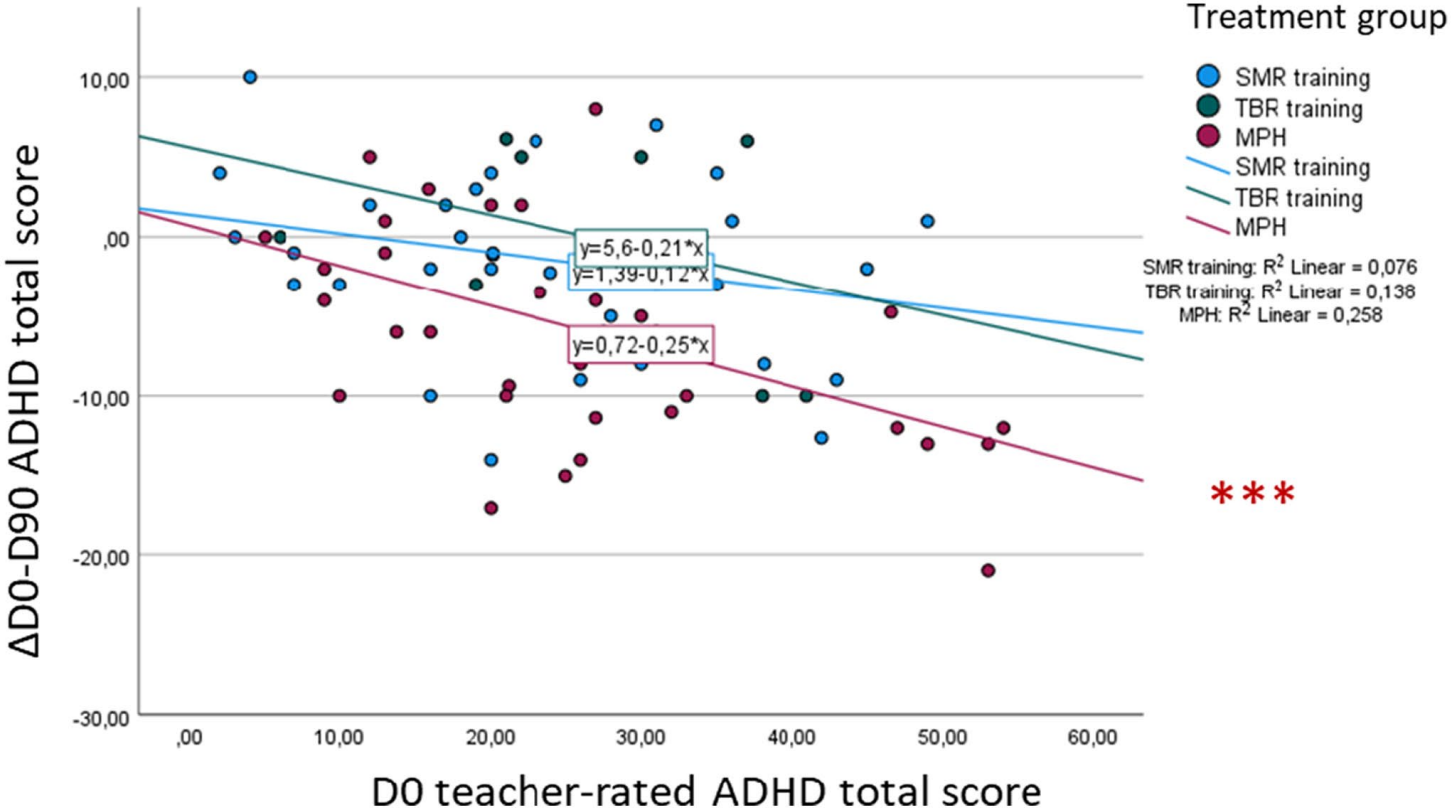
Association between baseline (D0) ADHD total score and D0-D90 symptom change—teacher rating. *p < 0.05, **p < 0.01, ***p < 0.001.

Furthermore, our analyses revealed a significant effect of baseline on teacher-rated ADHD total score symptom change [*F*(1, 81) = 14.39, *p* < 0.001]. *Post-hoc* analyses showed a significant correlation only in the MPH group (*r* = 0.63, *p* < 0.001), with no significant difference in correlations compared to the other groups (Figure 6).

## 4 Discussion

The aim of these analyses within the NEWROFEED study was to explore neurocognitive and higher-order EF indices as markers of treatment effectiveness and for predicting treatment response in children and adolescents with ADHD. The focus was on non-pharmacological, home-based (NF@Home) as well as pharmacological (treatment with MPH) treatment response.

With regard to treatment effectiveness NF@Home and MPH in general, our replication analyses for three treatment groups (additionally differentiating between the two different NF@home groups: SMR and TBR) showed that MPH treatment is significantly more effective compared to both NF@home groups. This supports the finding of inferiority of NF@home treatments in line with the previously published main analyses differentiating between two groups (MPH and NF@Home) combining both NF@Home training groups (64). This pattern of result could already be shown at D60, indicating a statistically significant difference in symptom improvement between the MPH group and the two NF@Home groups, respectively, already after a rather short treatment period of around 6 weeks. However, for all treatment groups a significant within-group effect was revealed pointing out to the effectiveness of all implemented treatments (MPH treatment, as well as both NF@Home trainings) conducted within the current study (over time) as rated by clinicians. For parent and teacher ratings, a similar pattern of results was obtained, but with weaker effects for both NF@Home groups and even non-significant within-group effects for the TBR training and a non-significant results for the SMR training for teacher-rated symptom levels (61, 62). In line with previous primary studies and meta-analytic findings, these results highlight that NF – also when conducted in the at home setting – might have less impact on less proximal symptom-level ratings and especially on behavior within the school setting. Importantly, clinician and parents ratings were not blinded in the current study (but teachers were not directly informed about treatment allocation). Compared to clinicians and parents, teachers might be less affected by positive expectation effects related to any kind of treatment. Therefore, their ratings could be considered as probably blinded (56). Nevertheless, expectancy effects might be relevant in both, parents and teachers (57). Smaller, even non-significant effects for teacher ratings have been revealed in previous meta-analytic findings as well (55, 56). One explanation might be that teachers are less sensitive to ADHD-symptoms variation than parents rather than being more objective (58, 59). However, recent findings challenge this assumption (60). Rather, lower symptom-level scores at baseline in teacher ratings could potentially contribute to the smaller within-group effects observed in our sample and in other studies (54).

Furthermore, with regard to the identification of treatment markers (cognitive indices sensitive to treatment effects and change for treatment monitoring), we found treatment effects on a variety of neuropsychological, higher-order EF indices, especially in the MPH group. In the MPH group, a significant effect of treatment was revealed for all neuropsychological, higher-order cognitive indices explored within this study. For the NF@Home training groups, only for the SMR training group a significant treatment effect on the BRIEF global score could be identified. These results are in line with previous studies, indicating robust effects of stimulant treatment on cognitive functioning indices (25, 27, 34, 35). Effects of NF treatment on complex EF skills appear less robust, comparably to earlier findings (29, 36). For the association between change in neuropsychological, higher-order EF indices and change in ADHD symptom levels, we found a robust correlation between change in symptom levels and the BRIEF global score across all raters. Further associations of changes were found for CPT commission errors in parent ratings only, as well as CPT hit RT variability in clinician and parent ratings. These findings indicate that changes on a parent-rated, behaviorally-focused scale are more closely linked to ADHD symptom changes (that are also rated on a behavioral level by a clinician) and that this association is not rater-dependent, contrary to the findings of Aggensteiner et al. (38) which showed that behavioral performance of the CPT task was associated with teacher ratings only (38). A robust association between clinical, behaviorally-based symptom changes and the BRIEF has been found in earlier studies. Again, no significant correlations were found for teacher ratings besides reflecting effects that are less proximal to the treatment setting as discussed above, this might be due to lower teacher ratings of change for the ADHD symptom level resulting in a lower variance for the respective analyses and consequently a lower probability of identifying a significant effect. In line with Coghill et al. (24), we found that treatment led to improvement in both, symptoms and higher-order cognitive processes, especially in the MPH group; however, these improvements were rather not correlated (22, 24). These results indicate, that those who improved on a clinical symptom-level, not necessarily improved with regard to CPT performance, in parallel with results from earlier studies. However, the current study focused on the assessment of higher cognitive functioning indices, and the measurement of “lower” cognitive processes might probably have revealed further promising effects, in line with earlier studies, e.g., on memory processing (32). Nevertheless, these findings rather point out the need of probably broadening the focus of the assessment of treatment effectiveness to the inclusion of objective neurocognitive markers (22) due to the highly relevant results indicating a close link between those neurocognitive indices and daily-life functioning rather than clinical symptoms (21).

With regard to prediction, results of the current study indicate a rather limited prognostic value of neuropsychological, higher-order cognitive functioning indices for treatment response to either MPH or NF@Home treatment. Only for parent-ratings, significant effects were revealed, pointing out a rater-dependency of the sensitivity of those markers limiting recommendations for clinical use Aggensteiner et al. (38) also found a significant predictive effect in parent-ratings; however, they revealed a significant effect for CPT commission errors, contrary to our results. Further, (interaction) effects found in this study are mainly driven by the small TBR group and should therefore be interpreted with caution. The small size of this subgroup represents the main shortcoming of the current trial. Based on these results, conclusions on differential treatment effects comparing either MPH or SMR training to TBR training should not be too enthusiastic and further studies are warranted for more solid recommendations. One interesting finding of this study were significant negative correlations of the baseline BRIEF global score with changes in parent-rated inattention symptoms from baseline to D90 in both, the MPH and SMR groups highlighting a potential need of further exploring this index and its potential predictive value in future, in line with conclusions from earlier studies (39). Thereby, higher BRIEF scores indicative of higher levels of executive dysfunction are associated with higher levels of symptom change (D0-D90). Again, this finding might be linked to a probably higher association of such parent-reported, behaviorally-focused clinical scales compared to objective performance tests with clinical scores. Besides, baseline ADHD symptom levels were revealed as the strongest predictor of symptom change from D0 through D90, in line with findings from a vast amount of earlier studies. This finding underlines the validity of the current dataset and applied statistical models as this effect represents an important landmark effect in the field of psychotherapy and psychopharmacological treatment research.

Shortcomings of the current study were a small TBR group, and a significantly older SMR NF@Home training group compared to the two other groups. Further, neuropsychological, higher-order cognitive indices analyzed and presented here were *a priori* selected based on previous publications (e.g., (39)). Other indices also taking into account “lower” cognitive processes or combinations of indices might be explored as further promising treatment or predictive markers. Future studies are warranted at this stage, with even larger, balanced samples, also taking into account a broader range of non-pharmacological treatment options for more valid conclusions.

## 5 Conclusion

Current findings indicate that the neurocognitive indices explored within the current study show a rather limited prognostic value with regard to predicting treatment response to NF@Home and MPH therapies questioning their usefulness in daily clinical practice. Baseline symptom severity was the most relevant predictor for treatment response, replicating robust findings from previous studies. However, results highlight a potential value of neurocognitive and higher-order EF indices as treatment markers for stimulant medication treatment in children and adolescents with ADHD, in line with earlier studies. As a marker of treatment effectiveness, the BRIEF scale (subjectively rated by parents) might be a valuable assessment scale used in clinical practice and across different raters and settings. Further, objectively measured CPT commission errors and hit RT variability might be promising markers for treatment monitoring in ADHD that need further exploration within future studies. Further studies are especially relevant at this stage due to the findings indicating a close relationship between neurocognitive indices and daily-life functioning highlighting the need for broadening the focus of the assessment of treatment effectiveness to the inclusion of objective neurocognitive markers.

## Data availability statement

The raw data supporting the conclusions of this article will be made available by the authors, without undue reservation.

## Ethics statement

The studies involving humans were approved by the Research Ethics Boards at each of the participating centers. The studies were conducted in accordance with the local legislation and institutional requirements. Written informed consent for participation in this study was provided by the participants’ legal guardians/next of kin.

## Author contributions

AK: Conceptualization, Data curation, Formal analysis, Investigation, Methodology, Project administration, Visualization, Writing – original draft, Validation. PA: Writing – review & editing. HB: Conceptualization, Funding acquisition, Writing – review & editing. TR: Conceptualization, Funding acquisition, Visualization, Writing – review & editing. EA: Conceptualization, Funding acquisition, Writing – review & editing. YA: Data curation, Writing – review & editing. TB: Conceptualization, Funding acquisition, Supervision, Writing – review & editing. SaB: Investigation, Writing – review & editing. EB: Writing – review & editing. AB: Conceptualization, Funding acquisition, Software, Writing – review & editing. MD: Conceptualization, Funding acquisition, Writing – review & editing. RiD: Conceptualization, Funding acquisition, Writing – review & editing. ReD: Conceptualization, Funding acquisition, Writing – review & editing. AG: Writing – review & editing. AH: Investigation, Writing – review & editing. LM: Conceptualization, Funding acquisition, Software, Writing – review & editing. KM: Investigation, Writing – review & editing. CM: Writing – review & editing. OR: Writing – review & editing. FT: Investigation, Writing – review & editing. SW: Conceptualization, Funding acquisition, Writing – review & editing. AW: Investigation, Writing – review & editing. StB: Conceptualization, Funding acquisition, Investigation, Writing – review & editing. DP-O: Conceptualization, Funding acquisition, Methodology, Supervision, Writing – review & editing. DB: Conceptualization, Funding acquisition, Methodology, Supervision, Writing – original draft.

## Footnotes

^1^ https://clinicaltrials.gov/ct2/show/NCT02778360
