# Supplementary material for: Limited usefulness of neurocognitive functioning indices as predictive markers for treatment response to methylphenidate or neurofeedback@home in children and adolescents with ADHD
Source: Front Psychiatry. 2024 Jan 12;14:1331004. doi: 10.3389/fpsyt.2023.1331004 (PMC10836215; doi:10.3389/fpsyt.2023.1331004)
Supplement: Supplementary file 1 [file Data_Sheet_1.docx]

Supplementary Material

# Supplementary Figures and Tables

**NEWROFEED CONSORT FLOW diagram**

**Figure S1 NEWROFEED CONSORT study flow chart (Purper-Ouakil et al., 2021)**


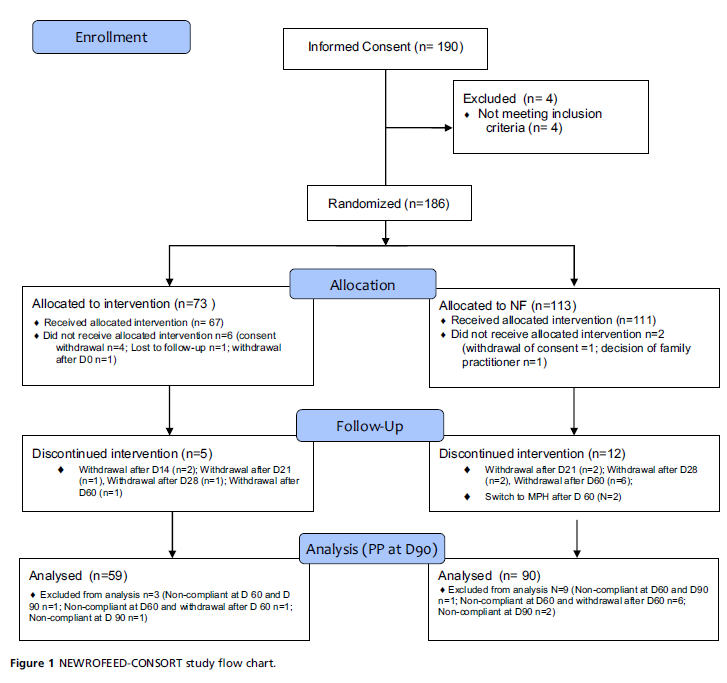


**Long ANCOVA results: between- and within-treatment effects: ADHD symptom change D0-D60-D90**

**Clinician ratings (D0-D60-D90)**

**Table S2**

*Clinician-rated between- and within-treatment effects: ADHD* ***total*** *score symptom change D0-D60-D90*

| **Index** | **Estimate (diff./change)** | **SE** | **95%CI** | **t** | **df** | **p** | **d** |
| --- | --- | --- | --- | --- | --- | --- | --- |
| **Within-group effects** |  |  |  |  |  |  |  |
| NF@Home SMR D0-D60 | -8.77 | 1.07 | -10.90-(-6.66) | -8.24 | 133 | <.001 | -1.04*** |
| NF@Home TBR D0-D60 | -9.16 | 1.95 | -13.00-(-5.30) | -4.69 | 133 | <.001 | -1.08*** |
| MPH D0-D60 | -16.00 | 1.20 | -18.40-(-13.60) | -13.3 | 133 | <.001 | -1.89*** |
| NF@Home SMR D0-D90 | -10.10 | 1.23 | -12.50-(-7.65) | -8.21 | 133 | <.001 | -1.19*** |
| NF@Home TBR D0-D90 | -6.67 | 2.36 | -11.40(-2.09) | -2.86 | 133 | <.01 | -.80** |
| MPH D0-D90 | -16.60 | 1.37 | -19.30-(-13.90) | -12.10 | 133 | <.001 | -1.97*** |
| **Between-group effects** |  |  |  |  |  |  |  |
| SMR vs. TBR (D60) | 0.39 | 1.91 | -3.39-4.17 | 0.21 | 133 | n.s. | .05 |
| SMR vs. MPH (D60) | 7.23 | 1.18 | 4.89-9.57 | 6.11 | 133 | <.001 | .86*** |
| TBR vs. MPH (D60) | 6.84 | 1.93 | 3.03-10.60 | 3.55 | 133 | <.01 | .81** |
| SMR vs. TBR (D90) | -3.31 | 2.41 | -8.08-1.45 | -1.38 | 133 | n.s. | -.39 |
| SMR vs. MPH (D90) | 6.55 | 1.49 | 3.60-9.50 | 4.39 | 133 | <.001 | .78*** |
| TBR vs. MPH (D90) | 9.87 | 2.44 | 5.04-14.70 | 4.05 | 133 | <.001 | 1.17*** |

*Note*. **p*<.05, ***p*<.01, ****p*<.001.

**Table S3**

*Clinician-rated between- and within-treatment effects: ADHD* ***inattention*** *score symptom change D0-D60-D90*

| **Index** | **Estimate (diff.)** | **SE** | **95%CI** | **t** | **df** | **p** | **d** |
| --- | --- | --- | --- | --- | --- | --- | --- |
| **Within-group effects** |  |  |  |  |  |  |  |
| NF@Home SMR D0-D60 | -5.40 | 0.61 | -6.59-(-4.20) | -8.91 | 133 | <.001 | -1.36*** |
| NF@Home TBR D0-D60 | -6.24 | 1.12 | -8.46-(-4.02) | -5.55 | 133 | <.001 | -1.57*** |
| MPH D0-D60 | -10.20 | 0.68 | -11.50-(-8.81) | 14.90 | 133 | <.001 | -2.56*** |
| NF@Home SMR D0-D90 | -6.05 | 0.67 | -7.38-(-4.72) | -9.01 | 133 | <.001 | -1.53*** |
| NF@Home TBR D0-D90 | -5.31 | 1.29 | -7.86-(-2.75) | -4.11 | 133 | <.001 | -1.34*** |
| MPH D0-D90 | -9.94 | 0.75 | -11.4-(-8.45) | -13.20 | 133 | <.001 | -2.51*** |
| **Between-group effects** |  |  |  |  |  |  |  |
| SMR vs. TBR (D60) | .84 | 1.10 | -1.33-3.02 | .77 | 133 | n.s. | .21 |
| SMR vs. MPH (D60) | 4.76 | 0.68 | 3.43-6.10 | 7.04 | 133 | <.001 | 1.20*** |
| TBR vs. MPH (D60) | 3.92 | 1.11 | 1.72-6.12 | 3.53 | 133 | <.01 | .99** |
| SMR vs. TBR (D90) | -.74 | 1.30 | -3.32-1.84 | -.57 | 133 | n.s. | -.19 |
| SMR vs. MPH (D90) | 3.89 | .80 | 2.30-5.48 | 4.85 | 133 | <.001 | .98*** |
| TBR vs. MPH (D90) | 4.63 | 1.32 | 2.02-7.24 | 3.51 | 133 | <.01 | 1.17** |

*Note*. **p*<.05, ***p*<.01, ****p*<.001.

**Table S4**

*Clinician-rated between- and within-treatment effects: ADHD* ***hyperactivity/impulsivity*** *score symptom change D0-D60-D90*

| **Index** | **Estimate (diff.)** | **SE** | **95%CI** | **t** | **df** | **p** | **d** |
| --- | --- | --- | --- | --- | --- | --- | --- |
| **Within-group effects** |  |  |  |  |  |  |  |
| NF@Home SMR D0-D60 | -3.49 | .61 | -4.70-(-2.27) | -5.69 | 133 | <.001 | -.53*** |
| NF@Home TBR D0-D60 | -3.37 | 1.14 | -5.61-(-1.12) | -2.96 | 133 | <.01 | -.51** |
| MPH D0-D60 | -5.99 | .69 | -7.37-(-4.62) | -8.64 | 133 | <.001 | -.91*** |
| NF@Home SMR D0-D90 | -4.14 | .68 | -5.49-(-2.79) | -6.07 | 133 | <.001 | -.63*** |
| NF@Home TBR D0-D90 | -1.90 | 1.31 | -4.49-0.70 | -1.45 | 133 | n.s. | -.29 |
| MPH D0-D90 | -6.84 | .77 | -8.36-(-5.32) | -8.90 | 133 | <.001 | -1.04*** |
| **Between-group effects** |  |  |  |  |  |  |  |
| SMR vs. TBR (D60) | -.12 | 1.12 | -2.33-2.10 | -.11 | 133 | n.s. | -.02 |
| SMR vs. MPH (D60) | 2.51 | .69 | 1.14-3.88 | 3.62 | 133 | <.01 | .38** |
| TBR vs. MPH (D60) | 2.63 | 1.13 | .40-4.85 | 2.33 | 133 | n.s. | .40 |
| SMR vs. TBR (D90) | -2.24 | 1.33 | -4.87-.40 | -1.68 | 133 | n.s. | -.34 |
| SMR vs. MPH (D90) | 2.70 | .82 | 1.07-4.33 | 3.28 | 133 | <.01 | .41** |
| TBR vs. MPH (D90) | 4.94 | 1.34 | 2.28-7.60 | 3.68 | 133 | <.001 | .75*** |

*Note*. **p*<.05, ***p*<.01, ****p*<.001.

**Parent ratings (D0-D60-D90)**

**Table S5**

*Parent-rated between- and within-treatment effects: ADHD* ***total*** *score symptom change D0-D60-D90*

| **Index** | **Estimate (diff.)** | **SE** | **95%CI** | **t** | **df** | **p** | **d** |
| --- | --- | --- | --- | --- | --- | --- | --- |
| **Within-group effects** |  |  |  |  |  |  |  |
| NF@Home SMR D0-D60 | -4.57 | 1.30 | -7.14-(-2.00) | -3.51 | 132 | <.001 | -.46*** |
| NF@Home TBR D0-D60 | -4.25 | 2.38 | -8.96-.46 | -1.79 | 132 | n.s. | -.43 |
| MPH D0-D60 | -12.80 | 1.46 | -15.70-(-9.91) | -8.76 | 132 | <.001 | -1.28*** |
| NF@Home SMR D0-D90 | -5.55 | 1.44 | -8.40-(-2.70) | -3.85 | 132 | <.001 | -.55*** |
| NF@Home TBR D0-D90 | -2.79 | 2.75 | -8.23-2.64 | -1.02 | 132 | n.s. | -.28 |
| MPH D0-D90 | -13.30 | 1.62 | -16.50-(-10.10) | -8.20 | 132 | <.001 | -1.33*** |
| **Between-group effects** |  |  |  |  |  |  |  |
| SMR vs. TBR (D60) | -.32 | 2.35 | -4.96-4.33 | -.14 | 132 | n.s. | -.03 |
| SMR vs. MPH (D60) | 8.23 | 1.47 | 5.33-11.10 | 5.62 | 132 | <.001 | .82*** |
| TBR vs. MPH (D60) | 8.55 | 2.35 | 3.89-13.20 | 3.63 | 132 | <.01 | .85** |
| SMR vs. TBR (D90) | -2.76 | 2.79 | -8.28-2.76 | -.99 | 132 | n.s. | -.28 |
| SMR vs. MPH (D90) | 7.75 | 1.74 | 4.30-11.20 | 4.45 | 132 | <.001 | .77*** |
| TBR vs. MPH (D90) | 10.50 | 2.81 | 4.94-16.10 | 3.74 | 132 | <.001 | 1.05*** |

*Note*. **p*<.05, ***p*<.01, ****p*<.001.

**Table S6**

*Parent-rated between- and within-treatment effects: ADHD* ***inattention*** *score symptom change D0-D60-D90*

| **Index** | **Estimate (diff.)** | **SE** | **95%CI** | **t** | **df** | **p** | **d** |
| --- | --- | --- | --- | --- | --- | --- | --- |
| **Within-group effects** |  |  |  |  |  |  |  |
| NF@Home SMR D0-D60 | -2.81 | .71 | -4.21-(-1.40) | -3.94 | 132 | <.001 | -.54*** |
| NF@Home TBR D0-D60 | -3.35 | 1.31 | -5.94-(-.75) | -2.55 | 132 | <.05 | -.65* |
| MPH D0-D60 | -7.84 | .80 | -9.42-(-6.26) | -9.81 | 132 | <.001 | -1.52*** |
| NF@Home SMR D0-D90 | -3.26 | .77 | -4.77-(-1.74) | -4.25 | 132 | <.001 | -.63*** |
| NF@Home TBR D0-D90 | -2.49 | 1.46 | -5.37-.40 | -1.71 | 132 | n.s. | -.48 |
| MPH D0-D90 | -7.43 | .86 | -9.14-(-5.73) | -8.61 | 132 | <.001 | -1.44*** |
| **Between-group effects** |  |  |  |  |  |  |  |
| SMR vs. TBR (D60) | .54 | 1.29 | -2.02-3.10 | .42 | 132 | n.s. | .11 |
| SMR vs. MPH (D60) | 5.04 | .80 | 3.45-6.62 | 6.30 | 132 | <.001 | .98*** |
| TBR vs. MPH (D60) | 4.50 | 1.30 | 1.92-7.07 | 3.45 | 132 | <.01 | .87** |
| SMR vs. TBR (D90) | -.77 | 1.47 | -3.67-2.13 | -.52 | 132 | n.s. | -.15 |
| SMR vs. MPH (D90) | 4.18 | .91 | 2.38-5.98 | 4.59 | 132 | <.001 | .81*** |
| TBR vs. MPH (D90) | 4.95 | 1.49 | 2.01-7.88 | 3.33 | 132 | <.01 | .96** |

*Note*. **p*<.05, ***p*<.01, ****p*<.001.

**Table S7**

*Parent-rated between- and within-treatment effects: ADHD* ***hyperactivity/impulsivity*** *score symptom change D0-D60-D90*

| **Index** | **Estimate (diff.)** | **SE** | **95%CI** | **t** | **df** | **p** | **d** |
| --- | --- | --- | --- | --- | --- | --- | --- |
| **Within-group effects** |  |  |  |  |  |  |  |
| NF@Home SMR D0-D60 | -1.79 | .72 | -3.21-(-.37) | -2.50 | 132 | .01 | -.27** |
| NF@Home TBR D0-D60 | -1.21 | 1.32 | -3.82-1.41 | -.91 | 132 | n.s. | -.18 |
| MPH D0-D60 | -5.05 | .81 | -6.65-(-3.44) | -6.23 | 132 | <.001 | -.75*** |
| NF@Home SMR D0-D90 | -2.30 | .80 | -3.89-(-72) | -2.87 | 132 | <.01 | -.34** |
| NF@Home TBR D0-D90 | -.61 | 1.53 | -3.64-2.43 | -.40 | 132 | n.s. | -.09 |
| MPH D0-D90 | -5.81 | .90 | -7.59-(-4.03) | -6.46 | 132 | <.001 | -.86*** |
| **Between-group effects** |  |  |  |  |  |  |  |
| SMR vs. TBR (D60) | -.59 | 1.13 | -3.17-2.00 | -.45 | 132 | n.s. | -.09 |
| SMR vs. MPH (D60) | 3.25 | .81 | 1.65-4.86 | 4.00 | 132 | <.001 | .48*** |
| TBR vs. MPH (D60) | 3.84 | 1.30 | 1.27-6.41 | 2.96 | 132 | .01 | .57** |
| SMR vs. TBR (D90) | -1.70 | 1.56 | -4.79-1.39 | -1.09 | 132 | n.s. | -.25 |
| SMR vs. MPH (D90) | 3.51 | .97 | 1.59-5.43 | 3.62 | 132 | .001 | .52*** |
| TBR vs. MPH (D90) | 5.21 | 1.56 | 2.12-8.30 | 3.33 | 132 | <.01 | .77** |

*Note*. **p*<.05, ***p*<.01, ****p*<.001.

**Teacher ratings (D0 -D90)**

**Table S8**

*Teacher-rated between- and within-treatment effects: ADHD* ***total*** *score symptom change D0-D60-D90*

| **Index** | **Estimate (diff.)** | **SE** | **95%CI** | **t** | **df** | **p** | **d** |
| --- | --- | --- | --- | --- | --- | --- | --- |
| **Within-group effects** |  |  |  |  |  |  |  |
| NF@Home SMR D0-D90 | -2.35 | 1.41 | -5.15-.46 | -1.67 | 74 | n.s. | -.20 |
| NF@Home TBR D0-D90 | -2.01 | 3.01 | -8.00-3.98 | -.67 | 74 | n.s. | -.17 |
| MPH D0-D90 | -10.00 | 1.42 | -12.90-(-7.19) | -7.05 | 74 | <.001 | -.85*** |
| **Between-group effects** |  |  |  |  |  |  |  |
| SMR vs. TBR (D90) | -.33 | 3.39 | -7.08-6.42 | -.10 | 74 | n.s. | -.03 |
| SMR vs. MPH (D90) | 7.68 | 2.04 | 3.61-11.70 | 3.76 | 74 | <.001 | .65*** |
| TBR vs. MPH (D90) | 8.01 | 3.31 | 1.41-14.60 | 2.42 | 74 | <.05 | .68* |

*Note*. **p*<.05, ***p*<.01, ****p*<.001.

**Table S9**

*Teacher-rated between- and within-treatment effects: ADHD* ***inattention*** *score symptom change D0-D60-D90*

| **Index** | **Estimate (diff.)** | **SE** | **95%CI** | **t** | **df** | **p** | **d** |
| --- | --- | --- | --- | --- | --- | --- | --- |
| **Within-group effects** |  |  |  |  |  |  |  |
| NF@Home SMR D0-D90 | -1.49 | .80 | -3.08-.10 | -1.86 | 75 | n.s. | -.25 |
| NF@Home TBR D0-D90 | -1.34 | 1.73 | -4.79-2.10 | -.78 | 75 | n.s. | -.23 |
| MPH D0-D90 | -5.84 | .82 | -7.47-(-4.21) | -7.15 | 75 | <.001 | *-.98** |
| **Between-group effects** |  |  |  |  |  |  |  |
| SMR vs. TBR (D90) | -.14 | 1.94 | -4.01-3.73 | -.07 | 75 | n.s. | -.02 |
| SMR vs. MPH (D90) | 4.36 | 1.17 | 2.03-6.68 | 3.73 | 75 | .001 | .73*** |
| TBR vs. MPH (D90) | 4.50 | .91 | .70-8.29 | 2.36 | 75 | n.s. | .76 |

*Note*. **p*<.05, ***p*<.01, ****p*<.001.

**Table S10**

*Teacher-rated between- and within-treatment effects: ADHD* ***hyperactivity/impulsivity*** *score symptom change D0-D60-D90*

| **Index** | **Estimate (diff.)** | **SE** | **95%CI** | **t** | **df** | **p** | **d** |
| --- | --- | --- | --- | --- | --- | --- | --- |
| **Within-group effects** |  |  |  |  |  |  |  |
| NF@Home SMR D0-D90 | -.87 | .69 | -2.25-.52 | -1.25 | 76 | n.s. | -.12 |
| NF@Home TBR D0-D90 | -1.04 | 1.51 | -4.06-1.98 | -.69 | 76 | n.s. | -.14 |
| MPH D0-D90 | -4.27 | .72 | -5.70-(-2.84) | -5.95 | 76 | <.001 | -.58 |
| **Between-group effects** |  |  |  |  |  |  |  |
| SMR vs. TBR (D90) | .17 | 1.70 | -3.22-3.56 | .10 | 76 | n.s. | .02 |
| SMR vs. MPH (D90) | 3.40 | 1.02 | 1.37-5.44 | 3.33 | 76 | <.01 | .46 |
| TBR vs. MPH (D90) | 3.23 | 1.67 | -.09-6.55 | 1.94 | 76 | n.s. | .44 |

*Note*. **p*<.05, ***p*<.01, ****p*<.001.
